# Comparative Transcriptome Analysis of Slow-Twitch and Fast-Twitch Muscles in Dezhou Donkeys

**DOI:** 10.3390/genes13091610

**Published:** 2022-09-08

**Authors:** Yan Li, Qingshan Ma, Xiaoyuan Shi, Wenmin Yuan, Guiqin Liu, Changfa Wang

**Affiliations:** 1 College of Agronomy, Shandong Engineering Technology Research Center for Efficient Breeding and Ecological Feeding of Black Donkey, Liaocheng University, Liaocheng 252000, China; 2 Marine Biomedical Research Institute of Qingdao, Qingdao 266000, China

**Keywords:** transcriptome, skeletal muscle, fiber type, donkeys

## Abstract

The skeletal muscle fiber profile is closely related to livestock meat quality. However, the molecular mechanisms determining muscle fiber types in donkeys are not completely understood. In this study, we selected the psoas major muscle (PM; mainly composed of oxidative-type muscle fibers) and biceps femoris muscle (BF; mainly composed of glycolytic-type muscle fibers) and systematically compared their mRNA and microRNA transcriptomes via RNA-seq. We identified a total of 2881 differentially expressed genes (DEGs) and 21 known differentially expressed miRNAs (DEmiRs). Furthermore, functional enrichment analysis showed that the DEGs were mainly involved in energy metabolism and actin cytoskeleton regulation. The glycolysis/gluconeogenesis pathway (including up-regulated genes such as *PKM*, *LDHA*, *PGK1* and *ALDOA*) was more highly enriched in BF, whereas the oxidative phosphorylation pathway and cardiac muscle contraction (including down-regulated genes such as *LDHB*, *ATP2A2*, *myosin-7 (MYH7)*, *TNNC1*, *TPM3* and *TNNI1*) was more enriched in PM. Additionally, we identified several candidate miRNA–mRNA pairs that might regulate muscle fiber types using the integrated miRNA–mRNA analysis. Combined with the results of protein–protein interaction (PPI) analysis, some interesting DEGs (including *ACTN3*, *TNNT3*, *TPM2*, *TNNC2*, *PKM*, *TNNC1* and *TNNI1*) might be potential candidate target genes involved in the miRNA-mediated regulation of the myofibril composition. This study is the first to indicate that DEmiRs, especially eca-miR-193a-5p and eca-miR-370, and potential candidate target genes that are mainly involved in actin binding (e.g., *ACTN3*, *TNNT3* and *TNNC1*) and the glycolysis/gluconeogenesis pathways (e.g., *PKM*) might coregulate the myofibril composition in donkeys. This study may provide useful information for improving meat quality traits in Dezhou donkeys.

## 1. Introduction

Skeletal muscle consists of different types of muscle fiber, which may affect the biochemical characteristics of the muscle [1]. The major muscle fibers can be roughly divided into the slow-twitch type (type I) and fast-twitch type (type II) according to the differences in their metabolic and contractile characteristics. With regard to metabolism, slow-twitch fibers, which are also known as the oxidative muscle type, show a higher abundance of mitochondria and myoglobin and higher oxidative metabolism than fast-twitch fibers (glycolytic type) [2]. It is well established that muscle fiber composition is an important factor affecting meat quality, including meat color, tenderness and flavor [3,4]. In addition to meat quality in animals, muscle fiber composition is also strongly related to muscle health in animals [5]. Therefore, the control of muscle fiber composition is of special concern not only for meat quality regulation, but also for molecular diagnostics and therapeutics. It was reported that gene expression in eukaryotes is specific to each tissue [6]. Further, the amount of gene products that are made in the same tissue, as well as in other tissues that make up that product, regulates the expression of that gene [7]. Additionally, one of the basic activities in domestic animals is the study of genes and proteins related to economic traits and their study at the cellular level [8]. Thus, understanding the differential expression patterns of muscle fiber at the transcript level in domestic animals is of critical importance. Donkey meat is drawing research interest, mainly due to characteristics including its high protein content and low fat content [9]. Although some studies have reported the differences among the various muscle fiber types in terms of physiology and functionality, the molecular mechanisms and signaling pathways involved in determining muscle fiber types in donkeys remain largely unknown.

MicroRNAs (miRNAs), which are important regulators of gene expression at the post-transcriptional level, have also been reported to play an important role in skeletal muscle growth and development [10,11]. Moreover, studies have broadened our understanding of the role of some muscle-specific miRNAs in the regulation of muscle fiber type composition and specification by regulating the expression of muscle-fiber-type-specific genes and associated transcription factors in certain livestock [12,13,14,15]. For example, Wang et al. showed that miR-499-5p acts as a positive regulator of oxidative myofiber formation in pigs by silencing Sox6 mRNA expression and increasing *MyHC I* and *MyHC IIa* mRNA levels [16]. MicroRNA-23a has been observed to affect muscle fiber composition by repressing the expression of *MEF2c* and its downstream genes (including *PGC1-α*, *NRF1* and *mtTFA*) [17]. miR-143-5p, miR-499-5p and miR-129-3p have been shown to be involved in determining muscle fiber composition in chickens by regulating the CaN/NFAT signaling pathway [15]. Knowledge of the roles of miRNAs in controlling the donkey muscle fiber profile remains limited. In a recent report on horses, Bao et al. demonstrated that miR-499 and miR-206 can regulate the expression of *SOX6*, thereby affecting the performance of fast- and slow-twitch muscle fiber types [18].

The Dezhou donkey, an important breed in China, with a large size, is famous for its excellent characteristics (including its large size, muscular body and excellent skin quality) [19]. Past studies in our lab have found differences in meat quality among various muscles of Dezhou donkeys (such as the donkey gluteus vs. longissimus dorsi) [20], and Chai et al. identified some differentially expressed genes (DEGs) related to tenderness in donkey meat (including *MYH1*, *MYH7*, *MYH4* and *MYL2*) [9]. However, the regulation mechanisms underlying muscle fiber type specification in Dezhou donkeys at the transcript level still need to be further elucidated. To address this, we studied mRNA–miRNA interactions to further explore the difference between the two muscle fiber types. In this study, we compared the mRNA and miRNA differences between the psoas major muscle (PM; mainly composed of oxidative-type muscle fibers) and biceps femoris muscle (BF; mainly composed of glycolytic-type muscle fibers) and identified the regulatory mechanism of muscle fiber composition. Therefore, the study provides a deep understanding that may contribute to the quality control and improvement of donkey meat.

## 2. Materials and Methods

### 2.1. Animal Selection and Sample Collection

The trial was carried out at the Dezhou donkey original breeding farm authorized by Shandong Province (Dezhou city, Shandong, China) and all procedures used in this study were approved by the Institutional Animal Care Committee at Liaocheng University (Permit No. DFG21010103-1). Six healthy male donkeys with similar body weight (225 ± 6 kg; mean ± SEM) and aged 2.5 years were selected for this study. After a 12 h feed withdrawal, six donkeys were slaughtered for tissue collection, as was done in our previous publication [21]. After death, the neck muscle (NM), psoas major muscle (PM), longissimus doris muscle (LD) and biceps femoris muscle (BF) were sampled and washed with sterilized saline, and immediately placed in liquid nitrogen for histochemical analysis and RNA extraction. Then, all frozen samples stored in dry ice were transported to the laboratory and stored at −80 °C for further analysis.

### 2.2. Histochemical Analysis

Histochemical analyses were performed with ATPase staining using the method of Brooke and Kaiser [22] with slight modifications. Transverse serial sections of 10 µm in thickness were cut from entire blocks (1.0 × 1.0 × 1.5 cm) with a cryostat microtome (HM525, Microm GmbH, Walldorf, Germany) at −20 °C. Sections were subsequently used for histochemical analysis of myosin adenosine triphosphatase (mATPase) following alkaline (pH 10.70) preincubation. An image analysis system (Image-Pro^®^ plus 5.1, Media Cybernetics Inc., Rockville, MD, USA) was used to examine the stained sections. The muscle fibers were classified as fiber types I, IIA and IIB according to the nomenclature of Brooke and Kaiser. Approximately 500 fibers per sample were counted to analyze the muscle fiber characteristics with Image-Pro Plus software (Image-Pro^®^ plus 5.1, Media Cybernetics Inc., Rockville, MD, USA), including the fiber number percentage (FNP) and fiber area percentages (FAP). FNP shows the ratio of the counted fiber number of each fiber type to the total counted fiber number. FAP was the ratio of a total cross-sectional area of each fiber type to total fiber area measured.

### 2.3. RNA Extraction, Sequencing and Bioinformatics Analysis

Total RNA was extracted from PM and BF muscle samples of the donkeys using Trizol reagent (Invitrogen, Carlsbad, CA, USA) following the manufacturer’s protocol. RNA quality was verified using the Agilent 2100 Bioanalyzer (Agilent Technologies, Santa Clara, CA, USA) and the NanoDrop2000 (Thermo Scientific, Wilmington, NC, USA), and then high-quality RNA samples were used for library preparation and sequencing. The paired-end RNA-seq sequencing libraries were sequenced with the Illumina NovaSeq 6000 (2 × 150 bp read length) using 5 μg of total RNA. The RNA-seq data were analyzed with the free online platform Majorbio Cloud Platform (http://www.majorbio.com). Briefly, quantification of gene expression as transcript per million (TPM) values was carried out using the RSEM algorithm (Version 1.3.1) [23]. The differential expression genes (DEGs) were identified and screened by DESeq2 based on fold change > 1.5 and *p*-value < 0.05 [24].

### 2.4. Small RNA Sequencing and miRNA Analysis

The miRNA data were also analyzed with the free online Majorbio Cloud Platform (http://www.majorbio.com). The reads were mapped to the reference genome of *Equus_asinus* (https://www.ncbi.nlm.nih.gov/genome/7038?genome_assembly_id=1720012, accessed on 24 March 2022) and classified by alignment against Rfam 12.3 (http://rfam.janelia.org/) and miRBase 22.1 (http://www.mirbase.org/). The publicly available software miRDeep2 (https://www.mdc-berlin.de/content/mirdeep2-documentation) was used to identify novel and known miRNAs [25], and sequences matching *Equus_caballus* in miRBase were considered known miRNAs. As mentioned above, the miRNA expression levels were calculated and normalized to transcripts per million (TPM) values. Significantly differentially expressed miRNAs (DEmiRs) were identified and screened by using DEseq2 according to the criteria of a fold change > 1.5 and a *p*-value < 0.05. Finally, miRandaSoft (score cutoff ≥ 160.0, energy cutoff ≤ −20 kcal/mol, http://www.miranda.org/) and RNAhybrid (number of hits per target ≥ 100, energy cutoff ≤ −20 kcal/mol, *p*-value cutoff ≤ 0.01, http://bibiserv.techfak.uni-bielefeld.de/rnahybrid/) were used to predict the target genes of the DEmiRs.

### 2.5. Functional Enrichment Analysis

All DEGs and target genes of DEmiRs were used for functional enrichment analysis, including Gene Ontology (GO) and Kyoto Encyclopedia of Genes and Genomes (KEGG) pathway analysis, with the free online platform Majorbio Cloud Platform (http://www.majorbio.com). The results with *p*-adjust values less than 0.05 were considered significantly enriched.

### 2.6. Real-time Quantitative PCR

To validate the transcriptome results, six mRNAs and two miRNAs were randomly selected for qRT-PCR. The relative expression analysis of genes was assessed by qRT-PCR as described in our previous study [9]. Trizol reagent was used to extract the total RNA of muscle samples, and reverse transcription was carried out using the PrimeScript RT Reagent Kit (TaKaRa, Dalian, China). *GAPDH* gene was used as a housekeeping gene in this procedure to normalize the gene expression data. Real-time qPCR was performed using ChamQ SYBR Color qPCR Master Mix (2X) (Vazyme, Nanjing, China) in a 7300 Real-Time PCR System (Applied Biosystems, USA). For miRNA analysis, 1 µg of RNA was used for reverse transcription with Maxima Reverse Transcriptase (Thermo Fisher Scientific, Vilnius, Lithuania). The U6 snRNA was used as the internal reference. Real-time qPCR was performed using 2X SG Fast qPCR Master Mix (Bimake, Houston, TX, USA) in a QuantStudio 3&5 Real-Time PCR System (ABI/Thermo Fisher, Wilmington, NC, USA). The primers used in this study are listed in Table S1. The 2^–ΔΔCt^ method was used to quantitate mRNA and miRNA expression level.

### 2.7. Protein–Protein Interaction (PPI) Network Analysis

To further identify hub genes, a protein–protein interaction (PPI) network analysis was performed in the Search Tool for the Retrieval of Interacting Genes (STRING) database. In the network, each edge was obtained based on the edge weight score to quantify the interaction confidence, and the PPI network was constructed and visualized using Cytoscape software (v3.9.0, Cytoscape Consortium, San Diego, CA, USA).

### 2.8. Statistical Analyses

SPSS statistical software (Version 22, SPSS IBM, New York, NY, USA) was used for statistical analysis. Data on the muscle fiber characteristics of NM, LD, PM and BF muscle was assessed by one-way ANOVA followed by Duncan multiple comparison, and Student’s t test was used to analyze the differences in FAP and RT-PCR data between the PM and BF. The variability of results was expressed as the mean ± standard error. Means were considered significantly different at *p* < 0.05.

### 2.9. Data Availability

The dataset(s) supporting the conclusions of this article are available in the National Center for Biotechnology Information Sequence Read Archive (http://www.ncbi.nlm.nih.gov/sra) under BioProject accession numbers PRJNA870101 (RNA-seq data) and PRJNA870031 (small RNA-seq data).

## 3. Results

### 3.1. Muscle Fiber Type Populations

**Figure 1:**
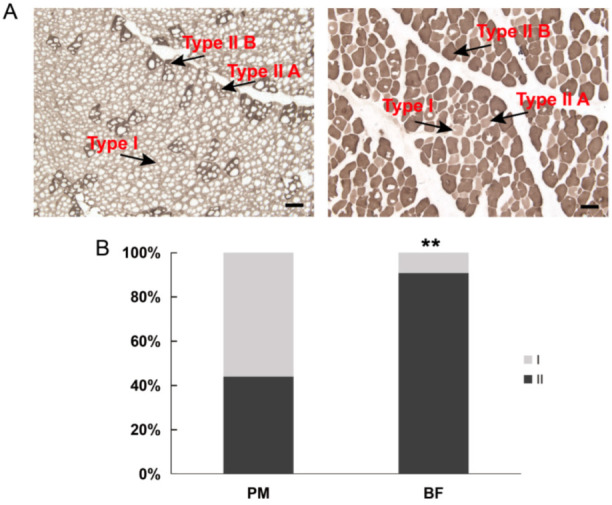
Muscle fiber type characterization in Dezhou donkeys. (**A**) Images of myosin ATPase staining (pH 10.7) of PM (**left**) and BF (**right**). The lightest muscle fibers are type I fibers, muscle fibers with an intermediate staining intensity are type IIA fibers and the darkest muscle fibers are type IIB fibers; bar = 100 μm. (**B**) Comparison of the muscle fiber type populations (%) between two muscles of donkeys. **: Significant differences between PM and BF (*p* < 0.001). I, type I; II, type II (type IIA and type IIB); PM, psoas major; BF, biceps femoris muscle.

Muscle fiber characteristics, including fiber diameter (FD), fiber number percentage (FNP) and fiber area percentage (FAP) are summarized in Table S2. In total, 24 samples (six sets of four muscles) were collected and analyzed, including the neck muscle, longissimus dorsi muscle from the back, psoas major muscle from the lower levels of the lumbar spine, and biceps femoris muscle from the hind limbs. As expected, a clear difference in the muscle fiber type composition was observed among the four muscle sections. The type I (slow) FAP of Dezhou donkeys was most prevalent in PM (55.99%), followed by NM (34.82%), LD (21.94%) and BF (9.22%) (Table S2 and Figure 1). Additionally, the diameter of the PM was thinner than that of the BF. Based on the above results, PM and BF were selected for the further study of muscle fiber types.

### 3.2. Overview of RNA and Small-RNA Sequencing

To assess the expression of mRNAs and miRNAs in the different muscle fiber types in donkeys, we collected PM and BF for the transcriptomic profiling of all mRNAs and miRNAs through the high-throughput sequencing of eight muscle samples (four each from PM and BF). In the mRNA-Seq libraries, a total of 504,369,474 raw reads were produced from the eight samples analyzed (Table S3) and deposited in the NCBI database under accession number PRJNA870101. After filtration, a total of 499,455,950 clean reads were obtained from the eight samples tested, more than 85% of which were mapped to the Dezhou donkey reference genome (Table S3). The Q20 and Q30 clean read quality scores of the eight samples were above 97% and 94%, respectively, indicating that the reliability and quality of the sequencing data met the standard analysis requirements and could be used for further analysis.

Additionally, in the small RNA-Seq libraries, an average of 11,452,819 clean reads were obtained from the eight samples. An average of more than 300 known miRNAs and 70 novel miRNAs were obtained after analyses and annotation (Table S4). Furthermore, the data supporting these results were deposited in the NCBI database under accession number PRJNA870031.

### 3.3. Differentially Expressed Genes (DEGs) between the Two Muscles

**Figure 2:**
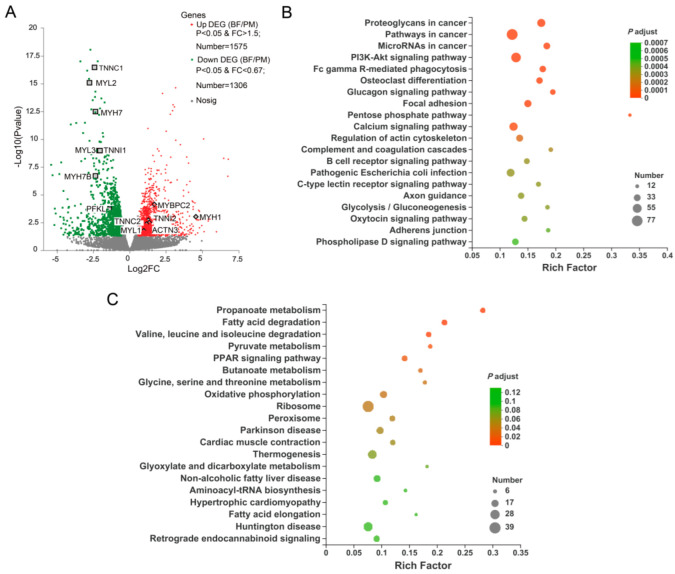
Differential RNA expression of data between PM and BF. (**A**) Volcano plots of DEGs; (**B**) top 20 significantly enriched KEGG pathways of up−regulated mRNAs in BF group; (**C**) top 20 significantly enriched KEGG pathways of down−regulated mRNAs in BF group. PM, psoas major; BF, biceps femoris muscle; DEGs, differentially expressed genes.

The PCA showed that the DEGs differed between the two muscle types of donkeys (Figure S1). Muscles were differentiated based on the presence of slow and fast myofibers. A total of 2881 differentially expressed genes (DEGs) were identified by comparing the gene expression between PM and BF, among which 1575 genes were up-regulated and 1306 genes were down-regulated in BF relative to PM (Figure 2A and Table S6a). The results indicated that there was a significant difference between the two muscle types at the mRNA level. As expected, BF showed higher expression levels of genes related to the fast-twitch muscle fiber type (up-regulated DEGs, *p* < 0.05), such as *TNNI2*, *TNNC2*, *MYH1*, *MYH2*, *ACTN3*, *MYBPC2* and *MYLPF*, whereas PM showed higher abundances of genes related to the slow-twitch muscle fiber type (down-regulated DEGs, *p* < 0.05), including *MYH7*, *MYH7B*, *TNNC1*, *TNNI1*, *MYL2* and *MYL3*.

To better understand the function of these DEGs, we performed further GO and KEGG pathway enrichment analyses (Figure 2B,C and Figure S2). After GO enrichment analysis, we observed that the DEGs were mainly enriched in biological pathways. Among the identified biological processes, the enriched GO terms were mainly related to muscle development and energy metabolism (Figure S2A). The results of the KEGG enrichment analysis also showed that the DEGs were enriched in some pathways involved in muscle profiles and function as well as energy metabolism, such as the glucagon signaling pathway, PPAR signaling pathway, glycolysis/gluconeogenesis, AMPK and PI3K-AKT signaling pathways, and regulation of actin cytoskeleton (Figure S2B). Additionally, the glycolysis/gluconeogenesis pathway (including up-regulated genes such as *PKM*, *BPGM*, *ENO2*, *LDHA*, *PGK1* and *ALDOA*) was more highly enriched in BF, whereas the oxidative phosphorylation pathway (including down-regulated genes such as *NDUFB3*, *NDUFC1*, *SDHB*, *ATP5MC1*, *NDUFB5* and *ATP5F1D*) was more enriched in PM (Figure 2B,C). In addition, some important genes involved in the calcium signaling pathway (such as *CAMK1*, *CALM1*, *CALML6*, *ANXA2* and *NFATC3* genes) were also identified in our study, and most of them were more highly expressed in BF.

### 3.4. Differentially Expressed miRNAs (DEmiRs) and Target Gene Prediction

To understand the expression characteristics of microRNAs in the slow-twitch PM and fast-twitch BF muscles, we constructed a small RNA library of the two muscles. After sequence alignment, 2528 known miRNAs and 621 novel miRNAs were identified (Table S4). A total of 21 known miRNAs were identified as DE-miRs between the two muscles (Table S5). Among these miRNAs, 7 miRNAs (such as eca-miR-199b-5p, eca-miR-370 and eca-miR-758) were up-regulated, and 14 miRNAs (such as eca-miR-196b, eca-miR-196a, eca-miR-192, eca-miR-615-3p, eca-miR-499-3p, eca-miR-128 and eca-miR-193-5p) were down-regulated in BF. Furthermore, 48,194 miRNA target genes were predicted and identified based on analyses with miRanda and RNAhybrid software. Among these genes, 1531 target genes were found to be previously identified DEGs, which were considered intersection genes (Table S6b).

### 3.5. qRT-PCR Validation

To validate the transcriptome results, the relative expression of five mRNAs (including *ACTN3*, *CPT1B*, *MYL1*, *MYL2* and *PFKL*) and two miRNAs (eca-miR-10a and eca-miR-758) was randomly selected for qRT-PCR quantification. As shown in Figure S3, the qRT-PCR expression patterns of these DEGs and two DEmiRs were in accordance with the results of the RNA-Seq analysis, indicating the reproducibility and reliability of our RNA-Seq results. Notably, *MYH4*, a marker of fast-twitch muscle fiber, was significantly more abundant in BF than in PM (Figure S3A), despite the failure to identify this gene in RNA-seq analysis.

### 3.6. Combined Analysis of DEGs and DEmiRs

**Figure 3:**
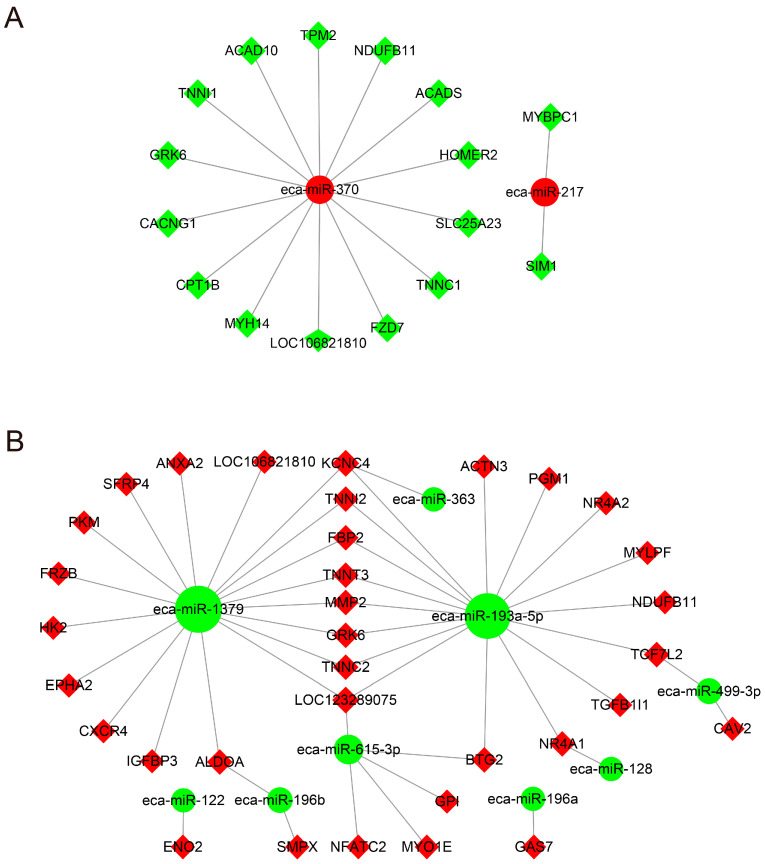
miRNA–mRNA association analysis. (**A**) Up-regulated miRNAs and down-regulated target genes related to the muscle fiber phenotype. (**B**) Down-regulated miRNAs and up-regulated target genes related to muscle fiber phenotype. Red nodes represent up-regulated miRNAs or genes, and green indicates down-regulated miRNAs or genes. Node size indicates the degree; a bigger node indicates a higher degree.

To reveal possible miRNA–mRNA interactions, we used Cytoscape mapping software (v3.9.0, Cytoscape Consortium, San Diego, CA, USA) to construct the potential miRNA–mRNA regulatory network. Based on GeneCards (www.genecards.org) and some references [6,26], more than 100 DEGs that might be closely related to muscle fiber composition were screened from the mRNA sequencing data (Table S6c). By considering these genes combined with the intersection genes, we screened 74 genes to construct a miRNA–mRNA regulatory network (Table S6d). We obtained 64 pairs of DEmiRs-DEGs, which included 9 down-regulated and 2 up-regulated miRNAs as well as 16 down-regulated and 48 up-regulated DEGs, and we then drafted the network of the DEmiRs and the DEGs based on the negative correlations (Figure 3). In the networks, eca-miR-193-5p, eca-miR-1379 and eca-miR-370 showed large numbers of target genes. Some important genes of the muscle fiber profile were found in this network, such as *ACTN3*, *TNNC2*, *TNNT3*, *MYPLF*, *TNNC1*, *NFATC2*, *PKM*, *TPM2* and *TNNI1*. Most genes that were up-regulated in BF were targets of the down-regulated miRNAs eca-miR-193-5p and eca-miR-1379 as well as eca-miR-615-3p (Figure 3B). On the other hand, *TNNC1*, *TPM2, TNNI1* and *MYH14* (also referred to as *MYH7b*), enriched in PM, were targets of the up-regulated miRNA eca-miR-370 (Figure 3A).

**Figure 4:**
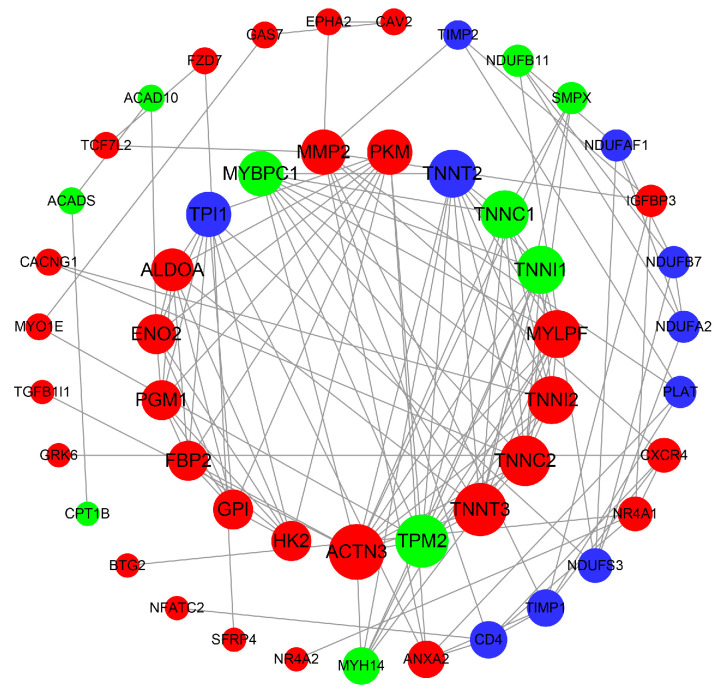
Protein–protein interaction (PPI) network of DEGs in the miRNA–mRNA network based on the STRING database. Red nodes indicate up-regulated genes, and green represents down-regulated genes. Node size indicates the degree; a bigger node indicates a higher degree.

Additionally, GO and KEGG enrichment analyses were carried out to explore the potential functions of these DEGs in this network. As shown in Figure S4A, the enriched GO terms were mainly related to biological processes including the Wnt signaling pathway, cell differentiation and glycolytic processes, cellular components such as troponin complex, and molecular functions such as actin binding. The enriched KEGG pathways were mainly associated with energy metabolism (such as glycolysis/gluconeogenesis and the pentose phosphate pathway), the Wnt signaling pathway, cancer (mainly related to cell proliferation and differentiation), and muscle contraction (Figure S4B). Finally, these genes in the miRNA–mRNA regulatory network were also used to build a PPI network, which included 272 edges and 50 nodes. The top 10 hub genes in network string interactions ranked by the degree method were *ACTN3*, *TPM2*, *TNNT3*, *TNNC2*, *MYLPF*, *TNNC1*, *TNNI1*, *TNNI2*, *PKM* and *MMP2* (Figure 4). The degree scores are shown in Table S6e.

## 4. Discussion and Conclusions

Skeletal muscle fiber is not only closely related to human muscle health but also affects meat quality in livestock production [1,4,15]. In our previous studies, we observed differences in meat quality among various muscles of donkeys (e.g., donkey gluteus vs. longissimus dorsi) [20] as well as genes related to donkey meat tenderness, as reported by Chai et al. [9]. However, the molecular mechanisms determining the muscle fiber profiles of donkeys remain unclear, especially with respect to miRNA regulation. Thus, we further explored the molecular mechanisms affecting muscle fiber types by comparing microRNA and mRNA differences through RNA sequencing of the two types of muscles. First, according to the observed muscle fiber characteristics, we selected PM and BF as representatives of type I muscle fibers (oxidative type) and type II muscle fibers (glycolytic type), respectively. Similar to previous reports, PM was characterized as the slow oxidative fiber type (type I), as found in pork [27,28], beef [29] and goat muscles [30], whereas BF showed higher respective percentages of type II fibers (fast-twitch fibers) [4,13,31]. Generally, the diameter of type I muscle fibers is thinner than that of type II muscle fibers [32], and our data were consistent with this pattern. Then, we identified a total of 2881 differentially expressed genes (DEGs) and 21 known differentially expressed miRNAs (DEmiRs) as well as several candidate miRNA–mRNA pairs that might regulate muscle fiber type by integrated miRNA–mRNA analysis.

In the present study, we also investigated the high functional divergence between PM and BF based on functional enrichment analysis. As expected, the functional analysis based on KEGG results showed that DEGs involved in energy metabolism, oxidative phosphorylation and cardiac muscle contraction were more enriched in PM, including some important genes (*LDHB*, *ATP2A2*, myosin-7 (*MYH7*), *MYH6*, *TNNC1*, *TPM3* and *TNNI1*) that are generally more highly expressed in slow-twitch skeletal muscle and cardiac muscle [18,33]. However, the expression of myosin-2 (*MYH2*), myosin-1-like (*MYH1*) and *MYH4*, which are molecular markers of adult fast/type II myofibers [34], was significantly higher in BF, indicating that BF was more enriched with type II myofibers than PM and showed a higher glycolysis level. Concerning the functional pathways of fast-twitch muscle fibers, BF was also mainly enriched in pathways related to the glucagon signaling pathway and glycolysis/gluconeogenesis, including the *PKM*, *PGK1*, *LDHA*, *PPP3CA*, *TNNC2* and *ALDOA* genes. For example, *PKM* is a key rate-limiting enzyme that catalyzes the last step in glycolysis [35], whereas *PGK* is involved in the first step in ATP generation. *PKM* has also been reported to participate in regulating meat quality; for instance, the expression of this gene is significantly negatively correlated with the final meat pH [28]. Another gene, *LDHA*, encodes skeletal-muscle-specific lactate dehydrogenase, which also plays an important role in glycolysis and affects the meat quality [36]. It is worth noting that most DEGs involved in the calcium signaling pathway, including *CALM1*, *CALM6*, *CALMK1*, *PPP3CA*, *ANXA2*, *NFATc2* and *NFATc3*, showed significantly higher levels in BF (mainly composed of fast-twitch muscle fibers), which was similar to the results of previous studies showing that the CaN/NFAT signaling pathway plays a key role in regulating the muscle fiber phenotype [15,37]. It has also been shown that the expression of *CALM1*, a subunit of CaM, is negatively correlated with intramuscular fat in pigs [38] and influences meat quality. Furthermore, genes such as *MYH1*, *MYH7*, *MYH4* and *MYL2* have been identified as candidate genes affecting meat tenderness in our previous work [9]. These results indicated that there is a difference between PM and BF at the transcription level, which is consistent with the data from the histochemical analysis of the study subjects. Additionally, some interesting genes may play an important role in regulating skeletal myofiber formation and thereby affect meat quality.

MicroRNAs can regulate target gene expression by promoting mRNA degradation or repressing translation. Several studies have also identified some differentially expressed miRNAs between glycolytic and oxidative muscles [13,15,16,17]. At the miRNA level, the present study is the first to identify a total of 21 known DEmiRs in comparisons of PM (mainly composed of slow-twitch muscle fibers) and BF (mainly composed of fast-twitch muscle fibers). Similarly, in Mongolian horses, only 11 microRNAs (including miR-499-3p, miR-499-5p and miR-206) were reported as DemiRs between the splenius (higher slow-twitch muscle fiber population) and gluteus medius (higher fast-twitch muscle fiber population) muscles [18]. Indeed, some of these DEmiRs have been reported to regulate muscle development and functions, including myofiber-type switching. For instance, miR-499 can affect myoblast differentiation and skeletal muscle fiber composition by repressing target genes such as *SOX6* and *FNIP* [16,18,39]. In turn, eca-miR-196a and eca-miR-196b were also more highly expressed in PM, whereas their expression was most absent in BF, which may be important for controlling myofiber type. In chickens, miR-196-5p is most enriched in slow-twitch muscle fibers, and can regulate myofiber type by targeting *CAML1* [15]. MiR-370-3p has been shown to down-regulate ACADSB gene expression and inhibit the formation of slow-twitch muscle fiber [40]. Additionally, some of the DEmiRs identified in our study, such as eca-miR-192, eca-miR-10a and eca-miR-128, are involved in muscle cell proliferation, differentiation and energy expenditure [41,42,43]. These results suggested that some DEmiRs identified in our study might also be involved in skeletal muscle fiber development and composition in donkeys.

To further understand the regulatory mechanism of miRNAs and their targets affecting the muscle fiber phenotype, an interaction network was constructed between the miRNAs and mRNAs involved in muscle development and muscle fiber composition. In this interaction network, some genes related to myofiber type pathways associated with the troponin complex (such as *TNNI2*, *TNNC2* and *TNNT3*), actin binding (*ACTN3*) and glycolysis/gluconeogenesis (such as *GRK6*, *PKM*, *EPHA*, *ALDOA* and *PGM1*), which are negatively regulated by eca-miR-193a-5p or/and eca-miR-1379, showed higher expression in BF (higher fast muscle fiber population). According to previous reports, miRNA-193a-5p mostly functions in the context of cell proliferation and differentiation, including tumor or cancer development and 3T3-L1 preadipocyte proliferation and differentiation [44,45,46]. For instance, the overexpression of miR-193a-5p can inhibit 3T3-L1 preadipocyte proliferation and differentiation by targeting *ACAA2* [46]. A study by Ju et al. showed that miR-193-3p may contribute to the regulation of oxidative myofibers in chickens by targeting the *PPARGC1A* gene [47]. Additionally, only one report has identified miR-1379 expressed in serum as a novel equine tissue miRNA, and the expression of this miRNA was relatively low in both muscles evaluated in our study, indicating that its regulatory role in muscle fiber composition in donkeys needs to be further confirmed. On the other hand, this study indicated that only one up-regulated miRNA showed a high degree in the interaction network. We found that some interesting genes (e.g., *TPM2*, *TNNC1*, *TNNI1*, *MYH14* and *ACADS*) were predicted to be targets of eca-miR-370, and these genes were more highly expressed in slow muscle fibers [18,40,48]. For instance, Zhang et al. revealed that miR-370-3p can inhibit the formation of slow-twitch myofibers by down-regulating the *ACADSB* gene, which might play an important role in fiber-type transitions [40]. Furthermore, combined with the results from PPI analysis, a number of potential candidate target genes affecting myofibril composition were screened, including *ACTN3*, *TNNT3*, *TPM2*, *TNNC2*, *PKM*, *TNNC1* and *TNNI1*. Considering these results together, we inferred that eca-miR-193a-5p and eca-miR-370 are very likely involved in controlling muscle fiber composition by targeting genes that are mainly involved in actin binding and the glycolysis/gluconeogenesis pathways in donkeys, although the cellular functional validation of these findings and the elucidation of the complex regulatory mechanisms involved will require further study.

In summary, we first compared the mRNA and miRNA transcript profiles of slow-twitch (PM) and fast-twitch (BF) muscles by RNA sequencing and constructed a miRNA–mRNA network. We identified 2881 DEGs and 21 DEmiRs between two muscles of Dezhou donkey. Furthermore, we identified two microRNAs (eca-miR-193a-5p and eca-miR-370) and some potential candidate target genes (*ACTN3*, *TNNT3*, *TPM2*, *TNNC2*, *PKM*, *TNNC1* and *TNNI1*), which were mainly involved in actin binding and the glycolysis/gluconeogenesis pathways, and might coregulate the muscle fiber types. Therefore, these miRNAs and potential candidate target genes could be used as candidate biomarkers controlling myofibril composition, and this study may expand our understanding of the molecular mechanisms underlying meat quality traits in donkeys.

## Data Availability

The dataset(s) supporting the conclusions of this article are available in the National Center for Biotechnology Information Sequence Read Archive (http://www.ncbi.nlm.nih.gov/sra, accessed on 16 August 2022) under BioProject accession numbers PRJNA870101 and PRJNA870031.

## Supplementary Materials

The following supporting information can be downloaded at: https://www.mdpi.com/article/10.3390/genes13091610/s1, Figure S1: Principal component analysis (PCA) based on RNA sequencing analysis of PM and BF. PM, psoas major; BF, biceps femoris muscle; Figure S2: Top 20 significantly enriched GO terms of the DEGs (A) and top 20 significantly enriched KEGG pathways of DEGs (B); Figure S3: Validation of differentially expressed mRNAs and miRNAs by RT-qPCR. (A and B) Validation of mRNAs by qRT-PCR. Five differentially expressed genes (*ACTN3*, *CPT1B*, *MYL1*, *MYL2* and *PFKL*) were randomly selected for qRT-PCR validation, and *GAPDH* was chosen as the reference gene. Additionally, the expression of *MYH4*, an important marker of fast-twitch muscle fiber, was also quantified by RT-qPCR, although this gene was not identified in the mRNA sequencing data in this study. (C and D) Validation of miRNA by qRT-PCR. Two differentially expressed miRNAs (eca-miR-10a and eca-miR-758) were randomly selected for qRT-PCR validation, and U6 snRNA was used as the internal reference. PM, psoas major; BF, biceps femoris muscle. Data are expressed as the means ± SEMs. a, b Means with different letters are significantly different, *p* < 0.05; Figure S4: Top 20 significantly enriched GO terms (A) and KEGG pathways (B) of DEGs in the miRNA–mRNA network; Table S1: List of primers used for differentially expressed genes and differentially expressed miRNA validation; Table S2: Muscle fiber characteristics of Dezhou donkey NM, LD, PM and BF muscles; Table S3: Overview of the mRNA sequencing data; Table S4: Overview of the small RNA sequencing data; Table S5: Differentially expressed miRNAs between PM and BF; Table S6: (a) DEGs in BF vs. PM (Up DEG (BF/PM), *p* < 0.05 & FC > 1.5; Number = 1575; Down DEG (BF/PF), *p* < 0.05 & FC < 0.67; Number = 1306); (b) Combined DEGs and predicted target genes of DEmiRs as the intersection genes (number = 1531); (c) DEGs related to the muscle fiber phenotype (number = 113); (d) Combined intersection genes and DEGs related to the muscle fiber phenotype; (e) Degree scores of the PPI network of the DEGs in the miRNA–mRNA network based on the STRING database.
