# Supplementary material for: Comparative Transcriptome Analysis of Slow-Twitch and Fast-Twitch Muscles in Dezhou Donkeys
Source: Genes (Basel). 2022 Sep 8;13(9):1610. doi: 10.3390/genes13091610 (PMC9498731; doi:10.3390/genes13091610)
Supplement: Supplementary file 1 [file genes-13-01610-s001.zip › Table S1.pdf]

Table S1. List of primers used for differentially expressed gene and differentially expressed miRNA validation.

Target mRNAs and miRNAs	Primers	Primer sequence (5'-3')	Product size (bp)
ACTN3	ACTN3 F	5' CATCACAGAAGCGGAACGG 3'	224
	ACTN3 R	5' CGCCAGGATCTTGAAGGAG 3'	
CPT1B	CPT1B F	5' GGCAACAGTGGGTTCCTC 3'	192
	CPT1B R	5' GGTCTGCGGTGTCTGGTAG 3'	
MYH4	MYH4 F	5' CACTTACCAGACTGAGGAGGAC 3'	164
	MYH4 R	5' ACCCGCAGCTTATTGACC 3'	
MYL1	MYL1 F	5' CTTTGTTGAGGGTCTGCG 3'	120
	MYL1 R	5' AGGGCTTCCACTTCTTCC 3'	
MYL2	MYL2 F	5' GGGGTGCTCAAGGCTGAT 3'	153
	MYL2 R	5' CCCGTGGGTGATGATGTG 3'	
PFKL	PFKL F	5' CCGAGGGTCCCGACTAAA 3'	201
	PFKL R	5' TCCATGCCCATCTTGCTG 3'	
eca-miR-10a	eca-miR-10a RT	5'CTCAACTGGTGTCGTGGAGTCGGCAATTC AGTTGAGCACAAATTCG 3'	69
	eca-miR-10a F	5' ACACTCCAGCTGGGTACCCTGTAGATCCG 3'	
	eca-miR-10a R	5' TGGTGTCGTGGAGTCG 3'	
eca-miR-758	eca-miR-758 RT	5'CTCAACTGGTGTCGTGGAGTCGGCAATTC AGTTGAGGGTTAGT 3'	66
	eca-miR-758 F	5' ACACTCCAGCTGGGTTTGTGACCTGGTCC 3'	
	eca-miR-758 R	5' TGGTGTCGTGGAGTCG 3'	
U6	U6 F	5' GTCTGCGGAAGCAAACCC 3'	102
	U6 R	5' AAGCCAGGACCCCTCGAT 3'	
GAPDH	GAPDH F	5' TGTCATCAACGGAAAGCC 3'	183
	GAPDH R	5' GCATCAGCAGAAGGAGCA 3'	
