# Supplementary material for: Comparative Transcriptome Analysis of Slow-Twitch and Fast-Twitch Muscles in Dezhou Donkeys
Source: Genes (Basel). 2022 Sep 8;13(9):1610. doi: 10.3390/genes13091610 (PMC9498731; doi:10.3390/genes13091610)
Supplement: Supplementary file 1 [file genes-13-01610-s001.zip › Table S2.pdf]

Table S2. Muscle fiber characteristics of Dezhou donkey NM, LD, PM and BF muscles.

Muscle fibercharacteristics	Muscle site			
	NM	LD	PM	BF
**FD/μm**				
I	86.87±2.69[a]	73.29±2.69[b]	34.99±0.75[d]	61.82±4.71[c]
IIA	78.47±5.20[a]	75.29±2.19[a]	31.27±1.95[b]	73.03±6.81[a]
IIB	67.02±4.32[b]	79.00±1.29[a]	32.75±1.96[c]	87.88±5.08[a]
**FNP/%**				
I	28.66±2.25[b]	23.79±1.74[b]	51.94±3.45[a]	15.04±1.84[c]
IIA	49.63±3.01[a]	37.01±0.66[b]	18.20±1.64[c]	45.35±1.46[a]
IIB	21.71±1.34[c]	39.28±1.06[a]	29.85±3.12[b]	39.60±0.62[a]
**FAP/%**				
I	34.82±1.07[b]	21.94±0.85[c]	55.99±5.19[a]	9.22±0.68[d]
IIA	48.49±2.63[a]	35.27±0.74[b]	16.03±2.40[c]	39.00±3.50[b]
IIB	16.69±2.30[d]	42.71±0.95[b]	27.98±3.13[c]	51.78±3.53[a]

Note: Values are expressed as means±standard error (SE). FAP, fiber area percentage; FD, fiber diameter; FNP, fiber number percentage; NM:neck muscle; LD, longissimus doris; PM: psoas major; BF: biceps femoris muscle. [a, b] Means within the same line with different superscript are significantly different, $p < 0.05$.
