# Supplementary material for: Comparative Transcriptome Analysis of Slow-Twitch and Fast-Twitch Muscles in Dezhou Donkeys
Source: Genes (Basel). 2022 Sep 8;13(9):1610. doi: 10.3390/genes13091610 (PMC9498731; doi:10.3390/genes13091610)
Supplement: Supplementary file 1 [file genes-13-01610-s001.zip › Table S3.pdf]

Table S3. Overview of the mRNA sequencing data.

Sample	Raw reads	Clean reads	Clean bases	Q20(%)	Q30(%)	Total mapped
BF1	61,740,332	61,220,666	9,063,013,666	98.09	94.44	53,434,252(87.28%)
BF2	60,075,304	59,535,278	8,774,272,974	98.1	94.46	52,882,130(88.82%)
BF3	65,277,766	64,597,352	9,501,792,219	97.91	94	57,304,502(88.71%)
BF4	65,595,026	64,984,792	9,572,760,141	98.05	94.33	57,949,251(89.17%)
PM1	57,910,162	57,335,852	8,458,740,962	97.98	94.14	49,582,344(86.48%)
PM2	64,470,662	63,848,432	9,363,539,518	97.96	94.11	56,009,453(87.72%)
PM3	61,777,260	61,168,262	9,011,198,704	98.02	94.29	55,799,100(91.22%)
PM4	67,522,962	66,765,316	9,784,468,516	97.95	94.1	57,271,906(85.78%)
Total	504,369,474	499,455,950	73,529,786,700	—	—	440,232,938
Mean	63,046,184	62,431,994	9,191,223,338	98.01	94.23	55,029,117
SEM	926,254	909,052	128,307,698	0.02	0.05	824,542
