# Supplementary material for: Comparative Transcriptome Analysis of Slow-Twitch and Fast-Twitch Muscles in Dezhou Donkeys
Source: Genes (Basel). 2022 Sep 8;13(9):1610. doi: 10.3390/genes13091610 (PMC9498731; doi:10.3390/genes13091610)
Supplement: Supplementary file 1 [file genes-13-01610-s001.zip › Table S4.pdf]

Table S4. Overview of the small RNA sequencing data.

Sample	Raw reads	Clean reads	Valid reads (18nt-32nt)	Known miRNA number	Novel miRNA number
BF1	12,366,699	12,236,054	11,843,951	326	82
BF2	11,886,550	11,840,244	11,681,790	303	65
BF3	12,334,223	12,204,647	11,882,111	323	82
BF4	10,559,200	10,475,261	10,176,843	312	73
PM1	10,508,034	10,403,243	10,118,409	318	76
PM2	12,356,741	12,186,428	11,637,666	321	83
PM3	12,487,755	12,346,296	11,941,376	313	74
PM4	10,057,443	9,930,376	9,640,297	312	86
Total	92,556,645	91,622,549	88,922,443	2,528	621
Mean	11,569,581	11,452,819	11,115,305	316.00	77.63
SEM	293,126	289,506	277,000	2.15	1.99
