# Supplementary material for: Comparative Transcriptome Analysis of Slow-Twitch and Fast-Twitch Muscles in Dezhou Donkeys
Source: Genes (Basel). 2022 Sep 8;13(9):1610. doi: 10.3390/genes13091610 (PMC9498731; doi:10.3390/genes13091610)
Supplement: Supplementary file 1 [file genes-13-01610-s001.zip › Table S5.pdf]

Table S5. Differentially expressed miRNAs between PM and BF.

miRNA	FC(BF/PM)[1]	Log2FC(BF/PM)	*P* value	Target genes[2]
Up-regulated				
eca-miR-217	15.586	3.962164973	0.010939969	227
eca-miR-758	2.236	1.161038807	0.004074418	270
eca-miR-136	1.98	0.985355114	0.013036673	165
eca-miR-148b-5p	1.655	0.726585871	0.024150124	127
eca-miR-199b-5p	1.574	0.653982491	0.02695249	363
eca-miR-409-3p	1.518	0.601801972	0.004190922	188
eca-miR-370	1.503	0.587547903	0.027945163	5537
Down-regulated				
eca-miR-196a	0.001	-10.00797814	6.03E-40	295
eca-miR-196b	0.003	-8.340656456	2.82E-41	610
eca-miR-208a	0.031	-5.032100466	0.004743204	22
eca-miR-615-3p	0.285	-1.813052283	0.00105634	1751
eca-miR-499-3p	0.368	-1.442579746	0.000957747	234
eca-miR-10a	0.384	-1.382136766	1.41E-09	192
eca-miR-122	0.47	-1.089913219	0.027859714	405
eca-miR-363	0.534	-0.904671244	0.021739573	106
eca-miR-9060	0.561	-0.834631743	3.81E-05	691
eca-miR-30e	0.605	-0.724508097	0.002704672	79
eca-miR-192	0.618	-0.693992312	0.038682673	94
eca-miR-128	0.637	-0.651728986	0.000290379	407
eca-miR-1379	0.646	-0.630577461	0.008971138	4440
eca-miR-193a-5p	0.656	-0.607369087	0.006353202	3342

[1]FC: Fold Change

[2]Target genes identified by miRanda and RNAhybrid.
