# Supplementary figures and images for: Comparative Transcriptome Analysis of Slow-Twitch and Fast-Twitch Muscles in Dezhou Donkeys
Source: Genes (Basel). 2022 Sep 8;13(9):1610. doi: 10.3390/genes13091610 (PMC9498731; doi:10.3390/genes13091610)

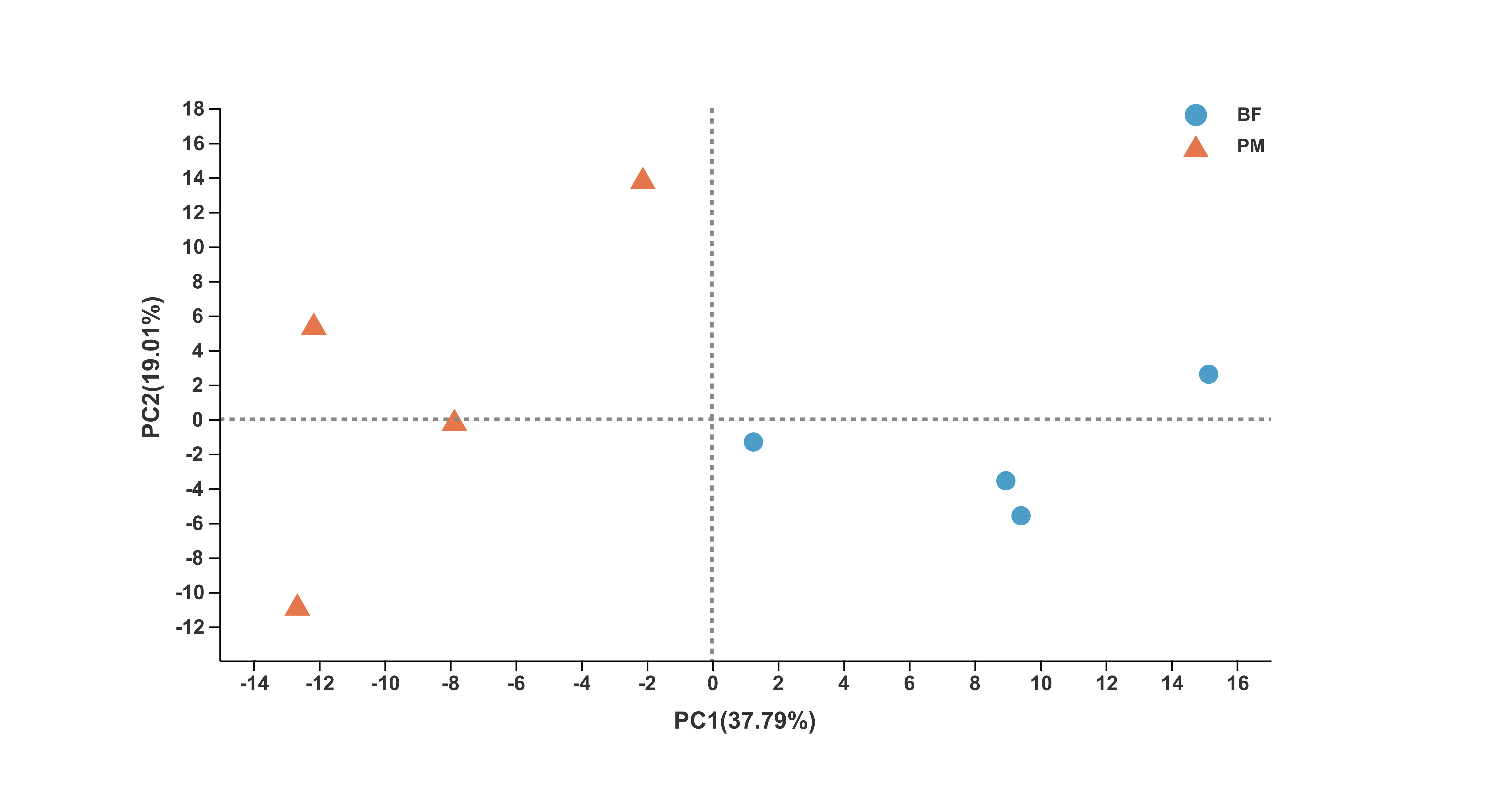

Supplement: Supplementary file 1 [file genes-13-01610-s001.zip › Figure S1.tif]

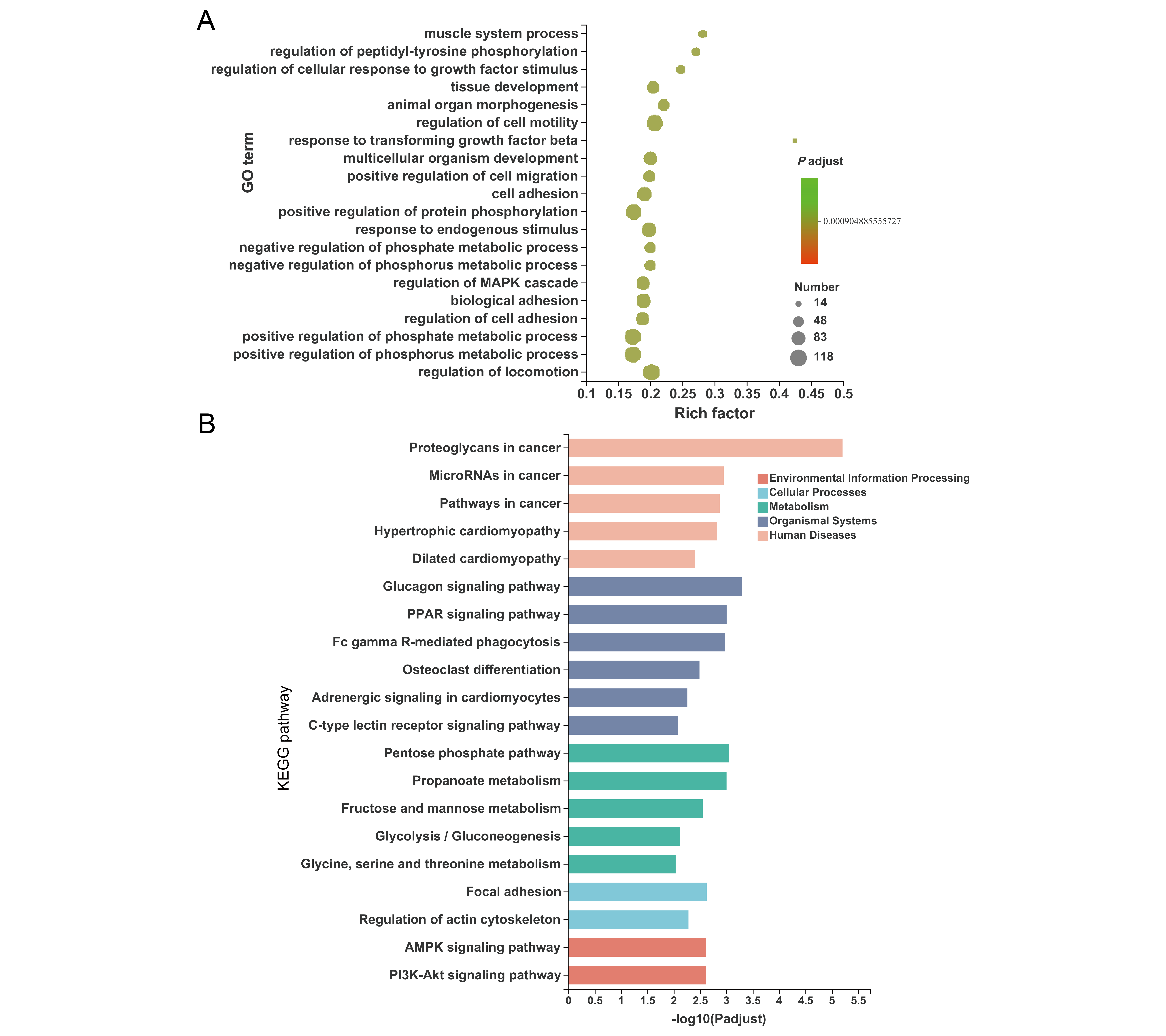

Supplement: Supplementary file 1 [file genes-13-01610-s001.zip › Figure S2.tif]

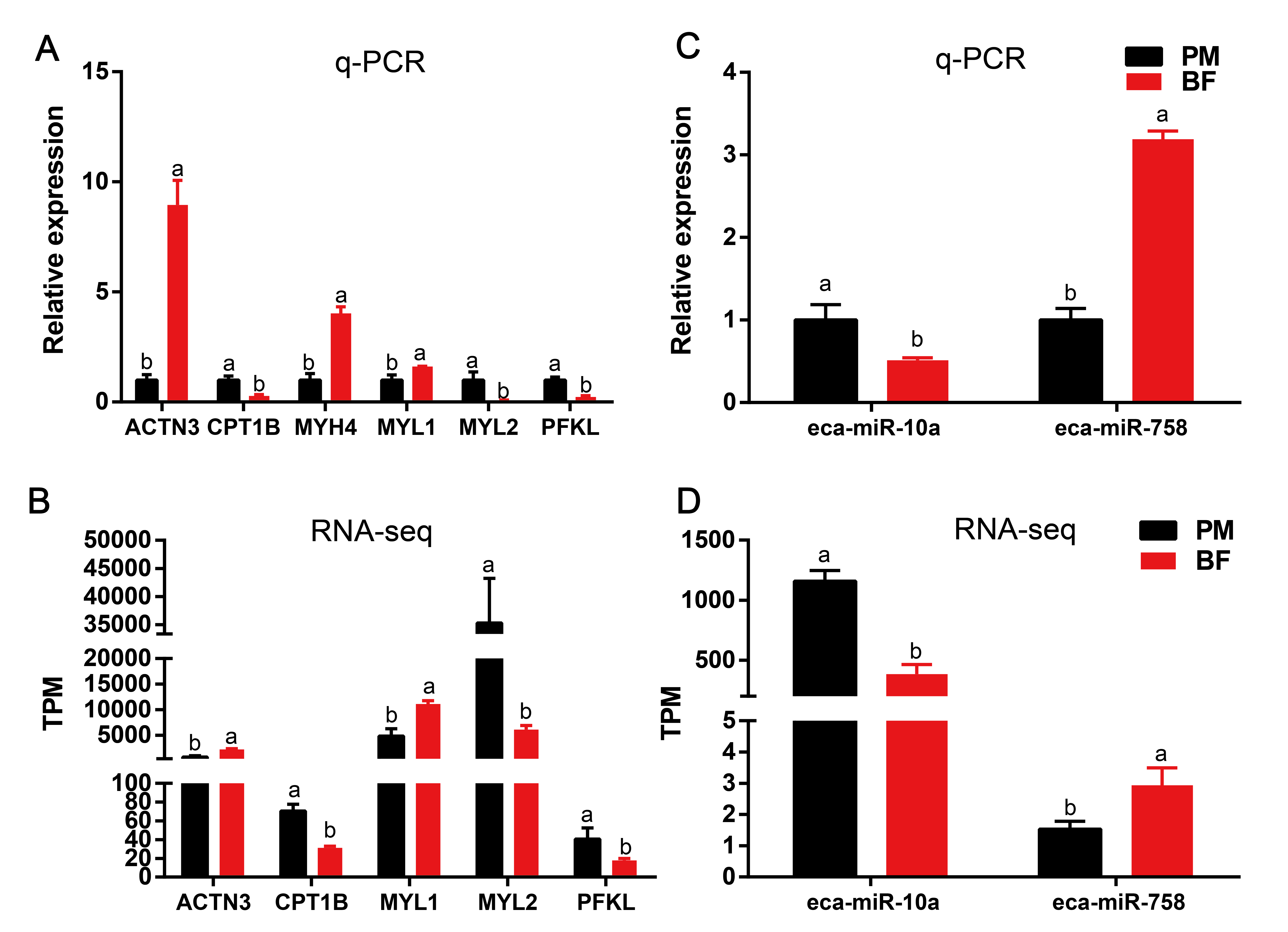

Supplement: Supplementary file 1 [file genes-13-01610-s001.zip › Figure S3.tif]

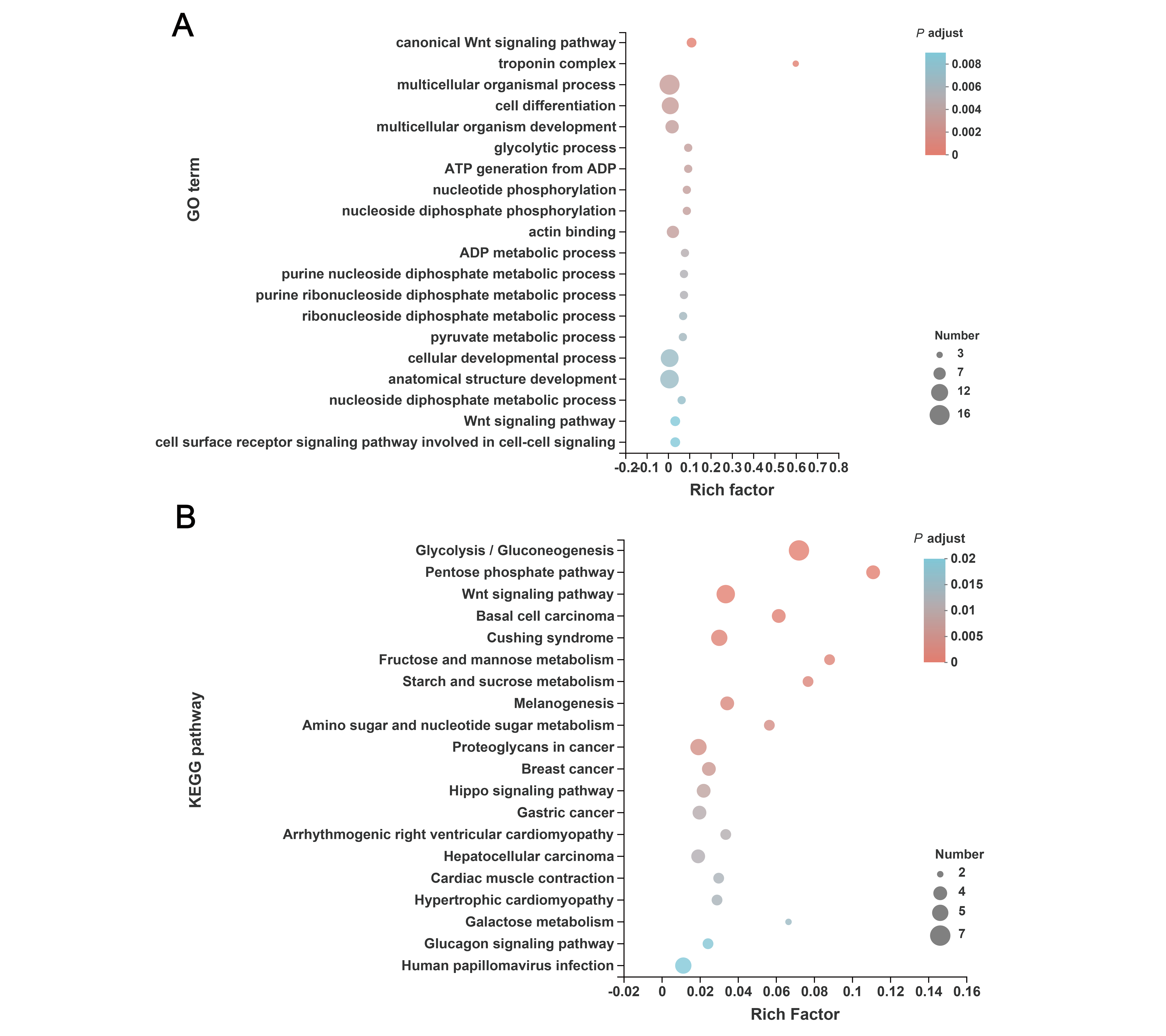

Supplement: Supplementary file 1 [file genes-13-01610-s001.zip › Figure S4.tif]
